# Leptin Promotes Fetal Lung Maturity and Upregulates SP-A Expression in Pulmonary Alveoli Type-II Epithelial Cells Involving TTF-1 Activation

**DOI:** 10.1371/journal.pone.0069297

**Published:** 2013-07-22

**Authors:** Hui Chen, Jian-Ping Zhang, Hui Huang, Zhen-Hua Wang, Rui Cheng, Wei-Bin Cai

**Affiliations:** 1 Department of Obstetrics and Gynecology, Sun Yat-sen Memorial Hospital of Sun Yat-sen University, Guangzhou, China; 2 Key Laboratory of Malignant Tumor Gene Regulation and Target Therapy of Guangdong Higher Education Institutes, Sun Yat-sen University, Guangzhou, China; 3 Department of Cardiology, Sun Yat-sen Memorial Hospital of Sun Yat-sen University, Guangzhou, China; 4 Department of Biochemistry, Zhongshan Medical School, Sun Yat-sen University, Guangzhou, China; 5 Center for Disease Model Animals, Sun Yat-sen University, Guangzhou, China; Virgen Macarena University Hospital, School of Medicine, Spain

## Abstract

The placental hormone leptin has important functions in fetal and neonatal growth, and prevents depressed respiration in leptin-deficient mice. The effect of leptin on respiratory distress suffered by low birth weight and premature infants has been studied. However, it is unclear how leptin enhances lung maturity in the fetus and ameliorates neonatal respiratory distress. In the present study, we found that antenatal treatment with leptin for 2 d significantly enhanced the relative alveolus area and improved the maturity of fetal lungs in a rat model of fetal growth restriction (FGR). Mean birth weight and lung wet weight were higher in the leptin-treated group than in the PBS-treated group, indicating promotion of fetal growth. Leptin upregulated the intracellular expression and extracellular secretion of surfactant protein (SP) A in type-II alveolar epithelial cells (AECs) *in vivo* and *in vitro*. Dual positive effects of leptin were found on protein expression and transcriptional activity of thyroid transcription factor-1 (TTF-1), a nuclear transcription essential for branching morphogenesis of the lung and expression of SP-A in type-II AECs. Knockdown of TTF-1 by RNA interference indicated that TTF-1 may play a vital role in leptin-induced SP-A expression. These results suggest that leptin may have great therapeutic potential for the treatment of FGR, and leptin-mediated SP-A induction and lung maturity of the fetus are TTF-1 dependent.

## Introduction

Fetal growth restriction (FGR), also known as fetal intrauterine growth retardation, refers to a condition in which a fetus has failed to achieve its genetically determined growth potential and affects up to 5–10% of pregnancies [1]. Neonates affected by FGR suffer from some pulmonary conditions, such as respiratory distress syndrome, neonatal asphyxia, pneumonia, and bronchopulmonary dysplasia, which are mostly due to fetal pulmonary immaturity and structural abnormalities, and a lack of surfactants [2], [3], [4]. Some endogenous and exogenous stimulators, such as glucocorticosteroids, have been shown to effectively enhance pulmonary maturity [5], [6]. Recently, studies have suggested that several cytokines and their receptors are localized in developing pulmonary tissues, and they play major roles in pulmonary epithelial cell differentiation and lung maturity [7], [8], [9], [10], [11]. Therefore, these cytokines and receptors have great therapeutic potential for the treatment of FGR.

Leptin, a 16-kDa peptide product of the *ob* gene, is a cytokine-like hormone primarily secreted from mature adipose tissue, as well as from many other tissues, such as the placenta, mammary epithelium, pituitary, gastric fundus, liver, and muscle [12], [13], [14]. The specific receptor for leptin is a member of the cytokine class I receptor superfamily, and is expressed in many tissues with multiple isoforms (Lep-Ra to Lep-Rf), which are generated by alternative splicing of the leptin receptor gene [15]. Leptin has pleiotropic effects on body weight, energy homeostasis, immune responses, inflammation, and angiogenesis [16], [17]. In recent years, a role of leptin in the regulation of respiration and pulmonary development has been discovered [8], [10]. Previous studies have demonstrated that leptin-deficient ob/ob mice suffer specific respiratory depression with alveolar hypoventilation and chronic hypercapnia [18], [19]. Leptin administration to ob/ob mice prevents respiratory depression and improves pulmonary diffusing capacity and lung compliance [20]. Moreover, in the fetus, the lung is one of the few tissues that express leptin. High-level expression of the functional leptin receptor and its splice variants has been detected in the fetal lungs, displaying specific binding for leptin [7], [21]. Isolated pulmonary type-II alveolar epithelial cells (AECs) from the fetus exhibit a unique response to leptin stimulation [7]. Recently, some studies have indicated that a lack of exposure to leptin late in pregnancy, when type-II AECs are maturing and producing surfactant, could contribute to the respiratory distress that occurs in FGR infants [8], [11]. Therefore, leptin may be an important regulator of pulmonary epithelial differentiation and lung development in the fetus [8], [10], [11], [17], [22].

However, how leptin enhances fetal lung maturity in physiological and pathological processes remains to be determined. Immaturity of fetal lungs is characterized by the production of an inadequate amount of surfactant proteinssuch as SP-A, SP-B and SP-C. SP-A, the most abundant protein component of lung surfactant is synthesized in pulmonary type-II AECs and plays a critical role in the formation of tubular myelin in the alveoli and pulmonary maturity [23], [24], [25], [26]. The expression of SP-A is regulated by thyroid transcription factor-1 (TTF-1), which affects morphogenesis and development of the lungs during early embryogenesis and at later stages of pregnancy [27], [28]. TTF-1 binding elements (TBEs) have been identified and characterized in the human SP-A gene, and the TBE core consensus sequence was found to be highly conserved and functionally critical for cAMP induction of SP-A promoter activity in Type-II AECs [29], [30].

In the present study, we investigated the potential for leptin to modulate pulmonary development, and attempted to determine the molecular basis and functional roles of leptin in pulmonary SP-A production and TTF-1 expression in a rat model of FGR.

## Materials and Methods

### Establishment of the FGR Rat Model and Treatment with Leptin

Sprague-Dawley rats, 6 to 8 weeks old, were obtained from the Experimental Animal Center of Sun Yet-san University (Guangzhou, China), and kept in a specific pathogen-free facility. The *in vivo* effect of leptin on fetal growth was studied in a rat model in which FGR was induced by partial uterine artery and vein ligation. Briefly, pregnant Sprague-Dawley rats were randomized into 3 groups with 10 animals per group on day 16 of gestation (term is 21.5 days), and anesthetized with 400 mg/kg of intraperitoneal chloral hydrate. The uterus was exposed under aseptic conditions and bilateral uterine vessel (artery and vein) partial ligation surgery was performed. The uterine vessels and a 3/0-nylon thread were ligated together by 1-silk suture, and then the nylon thread was removed for partial ligation. Validation of uteroplacental insufficiency was monitored by blood pressure of the uterine artery, which was monitored by a physical recorder. The uterus was returned to the abdominal cavity and the incision was closed. On days 19 and 20 of gestation, maternal rats received an intraperitoneal injection of 1 mg/kg body weight leptin (Sigma-Aldrich, St. Louis, MO, USA) or phosphate-buffered saline (PBS). The sham control group underwent identical anesthetic and surgical procedures, except for ligation. Caesarean section was performed on day 21. Birth weight, placental weight, and fetal lung weight were recorded. The respiratory score was performed as previously reported [31], [32]. Briefly, the pups were placed on moistened filter paper at 37°C immediately after removal from the mother. Breathing motion of each pup was observed for 1 minute by the same investigator, who was blinded to the treatment. Scoring assignment and respiratory pattern was determined according to the criteria described by Christensen HD [32] and Stewart JD [31]. The lungs of neonates were harvested and frozen in liquid nitrogen. Newborn pups were considered growth restricted when the initial body weight (iBW), measured within 24 h of birth, was below -2 SD of the mean iBW of pups in the control group.

### Ethics Statement

The study was approved by the Animal Care and Ethics Committee of SUN Yat-sen University, and all animal studies were performed under an institutionally approved protocol according to the guidelines and criteria from the committee (IACUC SYSU, NO.20061211015). All surgery was performed under chloral hydrate anesthesia, and all efforts were made to minimize suffering.

### Isolation and Culture of Type-II AEC Cells from Fetal Lungs

Type-II AECs were isolated and cultured as previously described by Cunningham et al., with some modifications [33]. Briefly, the 20-d pregnant rats were killed with an overdose of chloral hydrate (100 mg/ml) by intraperitoneal injection. The fetal lungs were removed aseptically and placed into ice-cold sterile PBS without calcium or magnesium under sterile conditions. After digestion with 0.05% trypsin and filtration through a 40 µm nylon mesh, the cell pellet was resuspended in DMEM containing 10% fetal bovine serum, and then added to rat IgG-coated 100-mm culture dishes (1.5 mg rat IgG/dish). After incubation for 30–60 min at 37°C in a CO_2_ incubator, the unattached cells were collected by centrifuging at 500 g for 5 min, and then resuspended in DMEM at a concentration of 1–2×10^7^ cells/ml. This cell suspension was cultured overnight in a culture flask or dish at 37°C in a CO_2_ incubator. Type-II AECs were identified by their appearance and lamellar body content in culture under phase-contrast microscopy, or by cytokeratin-positive staining. The isolated type-II AECs were cultured for 12 h before in vitro experiments were begun. For blocking test, an inhibitory leptin antibody (100 ng/ml, Y-20, from Santa Cruz Biotechnology, CA, USA) was added to type-II AECs with leptin. For hypoxic exposure in some experiments, type-II AECs were primarily cultured for 12 h and then cultured in an atmosphere of 0.5% oxygen, 5% CO_2_, and 94.5% nitrogen at 37°C for 12 h. This hypoxic atmosphere system was set up for simulating pathological hypoxia niche for cells *in vivo* [34].

### Plasmid Construction, Transient Transfections, and Luciferase Assay

The complete coding region of rat TTF-1 was amplified from type-II AECs by reverse transcription (RT)-PCR and cloned into *Bam*HI and *Kpn*I sites of pBIND (Promega, Madison, WI, USA) to make the pBIND-GAL4-TTF-1 construct. The pG5*luc* firefly luciferase reporter plasmid containing the five GAL4-binding sites upstream of the TATA box was obtained from Promega. The human alveolar epithelial A549 cells were maintained in F-12 nutrient mixture (Amersham Biosciences, Piscataway, NJ, USA) containing 10% fetal bovine serum at 37°C under 5% CO_2_. For transient transfection, plasmids were transfected into A549 cells using Lipofectamine 2000 (Invitrogen Carlsbad, CA, USA) according to the manual. The phRL-TK plasmid (Promega) was always cotransfected as an internal control for transfection efficiency. Six hours after transfection, the A549 cells were further cultured under serum-deprived conditions for 24 h with or without leptin. The transfected cells were harvested, lysed, centrifuged to pellet the debris, and subjected to luciferase assay. Luciferase activity was measured as chemiluminescence in a luminometer (PerkinElmer Life Sciences, Boston, MA, USA) using the Dual-Luciferase Reporter Assay System (Promega) according to the manufacturer’s protocol. All transfections were performed in triplicate and the results are expressed as means ± SEM of three independent experiments.

### Quantitative Real-time RT-PCR (qRT-PCR)

Total RNA was extracted from tissues of fetal lungs with TRIZOL reagent according to the manufacturer’s instructions (Invitrogen). The isolated RNA was reverse transcribed into cDNA using PrimeScript RT Master Mix (TaKaRa, Japan). Quantitative real-time RT-PCR was carried out using SYBR® Green Real-time PCR Master Mix (TOYOBO, Japan) in a Light Cycler System (Roche, Basel, Switzerland) with the following program: a denaturation step (95°C for 10 min) and 40 cycles of three-step amplification (denaturation, 95°C for 10 sec; annealing, 60°C for 10 sec; and extension, 72°C for 20 sec). All samples were amplified in duplicate, and β-actin was used as a reference gene for normalization. The following primers were used in the qRT-PCR assay (Table 1).

**Table 1:** Sequences of oligonucleotides used as forward and reverse primers for qRT-PCR.

Gene product	Forward primer	Reverse primer
leptin	5′-GACATTTCACACACGACGTC-3′	5′-GAGGAGGTCTCGCGAGTT-3′
leptin receptor	5′-ACCTTCAGTTCCAGATTCGA-3′	5′-TGAGATTGGTCTGATTTCCC-3′
SP-A	5′-AGCCTGCAGGTCTGTATGTGGA-3′	5′-TTGCACTTGATACCAGCGACAAC-3′
SP-B	5′-CCATCCCTCTGCCCTTCTG-3′	5′-CACCCTTGGGAATCACAGCTT-3′
SP-C	5′-TCCCAGGAGCCAGTTTCG-3′	5′-CACGATGAGAAGGCGTTTGA-3′
SP-D	5′-AAGTCATATGGGAAGGC-3′	5′-GGCCTGCCTGCACATCTC-3′
β-actin	5′-CACCCGCGAGTACAACCTTC-3′	5′-CCCATACCCACCATCACACC-3′

### Western Blot Analysis

Briefly, proteins (100 µg) prepared from the lysates of type-II AECs or fetal lung homogenization were separated by a 12% polyacrylamide gel, transferred to a polyvinylidene fluoride membrane, and subjected to western blot analysis with polyclonal antibodies against SP-A (H-148, Santa Cruz; diluted 1∶1000), SP-B (R-19, Santa Cruz; diluted 1∶500), SP-C (FL-197, Santa Cruz; diluted 1∶1000), SP-D (N-14, Santa Cruz; diluted 1∶500), leptin (Y-20, Santa Cruz; diluted 1∶500), leptin receptor (Sigma-Aldrich; diluted 1∶2000) or TTF-1 (H-190, Santa Cruz; diluted 1∶500) at 4°C overnight. After washing, the membranes were incubated with horseradish peroxidase-conjugated secondary antibodies, and visualized using an enhanced chemiluminescence detection kit (Pierce Biotechnology, Rockford, IL, USA) according to the manufacturer’s recommendations. The same membrane was stripped and reblotted with an antibody specific to β-actin (AC-15, Sigma-Aldrich, diluted 1∶10000). Specific band density was quantified by analysis of scanned images using ImagePro Plus 6.0 software (Media Cybernetics, Silver Spring, USA).

### Short Interfering RNA Silencing of TTF-1

Two TTF-1 short hairpin RNAs (shRNAs) (shttf-1a and shttf-1b) were designed, targeting the 19-nucleotide 5′-GGAGGAAAGCTACAAGAAA-3′ or 5′-GCTTCAAGCAGCAGAAGTA- 3′ in the coding sequence region of rat TTF-1 mRNA (NCBI accession number: XM_216720). To construct the shRNA expression vector, an oligonucleotide containing a sense 19-target sequence loop with TTCAAGAGA-antisense and its complementary sequence were synthesized. The complementary oligonucleotide pairs were annealed to generate double-stranded DNAs, and ligated into the linearized empty vector pBS/U6 (Ambion, Austin, TX, USA) at the *Apa*I and *Eco*RI sites. Plasmids (pBS/U6/shttf-1a and pBS/U6/shttf-1b) containing the correct insert were confirmed by DNA sequencing. An empty pBS/U6 vector was used as a negative control to detect nonspecific effects.

Type-II AECs were transfected with the plasmids constructed above by Lipofectamine 2000 (Invitrogen) according to the manufacturer’s instructions. After 6 h, the transfection medium was changed to DMEM, and cells were exposed to leptin for 24 h. Cell lysates were harvested, and the silencing efficiency was verified by western blotting.

### Histological and Immunohistochemical Analysis

Routine hematoxylin and eosin (HE) staining was performed for the lung tissues. The histological score and morphological features of fetal lung tissues were studied by light microscopy as an index for lung maturity. Histological score was performed by the pathologist who was blinded to the treatment according to the following lung development pattern and scoring system: 0 = pseudoglandular; 1 = canalicular; 2 = canalicular/terminal sac; 3 = terminal sac; 4 = terminal sac/alveolar; 5 = alveolar budding [31], [32].

Ten visual fields without blood vessels and bronchial tubes in each HE-stained section were randomly selected under a microscope (×200). Images of every visual field were captured by an Olympus microscope at ×200 magnification and transferred to an image analysis program (Image Pro Plus 6.0). Alveolar spaces were captured electronically, and the total alveolar area was obtained from the sum of individual alveolar air spaces within the selected microscopic field of vision. The area of the lung (Sp) and the airspace of the alveoli (Sa) were defined and calculated. The proportion of alveoli to lung tissue was presented by Sa/Sp. The alveolar septal thickness and tissue density, which is the proportion of the field occupied by alveoli and lung tissue, were analyzed and calculated by ImagePro Plus image analysis software [35].

For immunohistochemical analysis, the slides were blocked with endogenous peroxidase blocking solution and normal goat serum. After incubating with primary antibody for leptin (Y-20, Santa Cruz Biotechnology, 1∶50), Lep-R (Sigma-Aldrich, 1∶50), or TTF-1(H-190, Santa Cruz Biotechnology, 1∶100) at 4°C overnight, sections were incubated with a biotin-conjugated secondary antibody at room temperature for 30 min, and then incubated with enzyme conjugate (HRP-streptavidin). Immunostaining was revealed with streptavidin-peroxidase followed by the chromogenic substrate diaminobenzidine, and the sections were counterstained with hematoxylin. For negative-control staining, the primary antibodies were omitted.

### Immunofluorescence

Immunofluorescence was applied to determine the expression of TTF-1 in type-II AECs and carried out according to standard procedures. Briefly, cryosections were washed in PBS to remove OCT and then blocked at room temperature for 1 hour in blocking buffer (5% BSA and 0.1% Triton-X 100 in PBS). Sections were incubated in the primary antibody for 1∼2 hours. After washed 3 times (0.1% Triton-X 100 in PBS), samples were incubated in secondary antibody in the blocking buffer for 1 hour at room temperature. Sections were mounted with coverslips and DAPI was applied to detect nuclei. Fluorescent images were captured on an upright Leica fluorescent microscope.

Antibodies utilized in this study were as follows: rabbit anti-TTF-1 (1∶50, Santa Cruz), goat anti-SP-C (1∶50, Santa Cruz). Secondary antibodies: Alexa 594 Donkey anti-goat IgG (Invitrogen), Alexa 488 donkey anti-rabbit IgG (Invitrogen). The secondary antibodies were diluted at 1∶500 in blocking solution.

### Statistical Analysis

Experiments were routinely repeated three or more times, and data are presented as the mean ± standard error of the mean (SEM). Multiple group comparison testing was performed using SPSS 11.0 software (SPSS Inc., Chicago, IL, USA) and determined by one-way analysis of variance followed by the Student-Newman-Keuls post hoc test. A value of P<0.05 was considered statistically significant.

## Results

### Inhibition of the Leptin/Lep-R System in the FGR Rat Model

**Figure 1:**
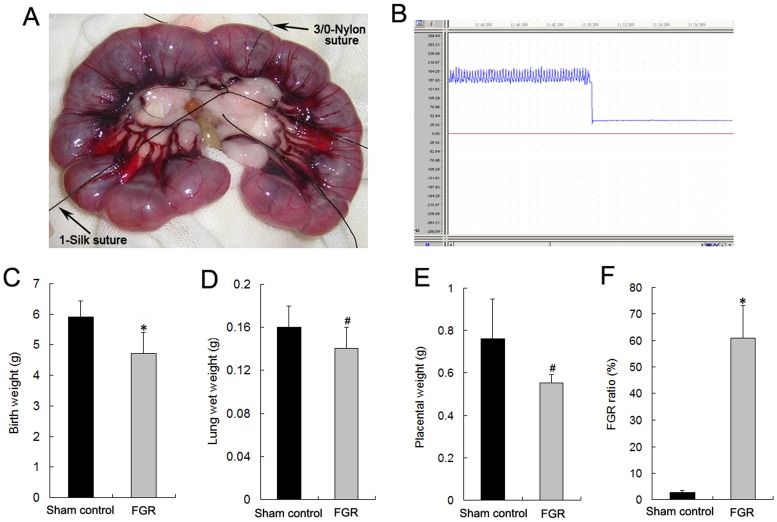
Establishment of the FGR rat model by partial ligation of bilateral uterine vessels. A and B: Bilateral uterine vessel partial ligation surgery was performed as per protocol (A) and blood pressure of the uterine artery was monitored by a physical recorder (B). C, D, and E: Birth weight (C), lung wet weight (D), and placental weight (E) of neonates were recorded for the assessment of FGR. *^#^P*<0.05 *vs* the sham control group (*n* = 10). F: In accordance with the criterion for FGR, the FGR ratio was determined by the number of FGR pups in relation to the total number of pups. ^*^P<0.01 *vs* the sham control group (*n* = 10).

The FGR rat model was established by partial ligation of the uterine arteries and veins at mid-gestation (day 16). As shown in Figure 1, birth weight, lung wet weight, and placental weight were lower in the neonatal pups of the FGR group compared with the sham control group. The mean iBW of pups in the normal pregnancy group was 5.91±0.52 g. Therefore, the criterion for FGR was an iBW <4.87 g. According to this criterion, the FGR ratio in the FGR group was significantly higher than that of the sham control group (Figure 1F, *P*<0.01).

**Figure 2:**
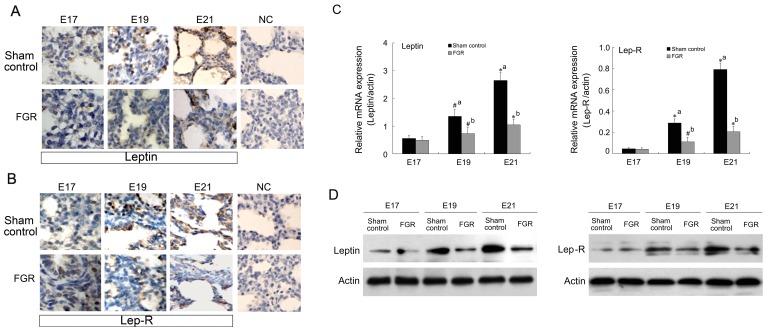
Expression of leptin and Lep-R in fetal lungs of the FGR rat. A and B: Leptin (A) and Lep-R (B) in fetal lungs at embryonic day 17 (E17), day 19 (E19) and day 21 (E21) were determined by immunohistochemical staining (×400). NC: the negative control. C: Fetal lung tissue from different stages of FGR rats was collected, and total RNA was extracted for mRNA expression measurement of leptin (right) and Lep-R (left) by quantitative real-time RT-PCR. ^*^P<0.01, *^#^P*<0.05; a: *vs* E17 sham group, b: *vs* the corresponding sham group (*n* = 3). D: Fetal lung tissue isolated from different stages of FGR and sham control rats were subjected to western blot analysis to determine the protein levels of leptin and leptin receptor. All data were shown as means of three independent experiments.

We then investigated the expression level of leptin and Lep-R in fetal lung tissues of FGR rats. Microscopic images of immunohistochemistry showed positive staining for leptin and Lep-R in pulmonic stroma and epithelial cells. Although leptin and Lep-R in the sham control and FGR groups gradually increased with gestational age, immunoreactant intensity of leptin and Lep-R in the FGR group appeared weaker than that in the sham control group on embryonic days 17, 19, and 20 of gestation (Figure 2A, 2B). Moreover, mRNA levels of leptin and Lep-R were significantly lower at different stages of late pregnancy in the FGR model than in the sham control group (Figure 2C). In addition, similar change pattern of leptin and Lep-R protein in the whole fetal lung tissues from FGR or sham control rats was confirmed by western blot analysis (Figure 2Dhttp://www.jimmunol.org/content/172/3/1809.full - F1#F1). We found that the leptin/Lep-R system was expressed in the fetal lungs and was detected at lower levels in the FGR group than in the sham control group.

### Leptin Promotes Fetal Growth and Lung Maturity in the FGR Rat Model

To evaluate the effect of leptin on fetal growth, maternal rats in the FGR model received an intraperitoneal injection of leptin or PBS in the late stage of gestation. As shown in Tables 2 and 3, the mean birth and lung wet weights in the leptin-treated group were greater than those in the PBS-treated group, which indicated a positive effect of leptin on fetal growth of pups in the FGR model. According to the criterion for FGR, the FGR ratio of the leptin-treated group was significantly lower than that of the PBS-treated FGR group (*P*<0.01, Table 2). These findings showed that leptin, at a dose of 1 mg/kg in late pregnancy, significantly promoted fetal growth in the FGR rat model.

**Table 2:** Effect of leptin on the FGR ratio, birth weight, and placental weight of neonatal rats.

Group	Number of neonates	Birth Weight (g)	Placental Weight (g)	FGR Ratio (%)
Sham control	72	5.91±0.52*	0.76±0.19#	2.77±0.65*
FGR+PBS	57	4.70±0.71	0.55±0.04	60.78±12.5
FGR+leptin	50	5.09±0.45#	0.56±0.09	27.14±4.4*

The number of neonates was recorded, and the weights of the placenta and the birth weight of the baby were measured within 24 hour after delivery. Newborn pups were considered with growth restriction when the iBW was below -2 SD of the mean iBW of the pups in the control group. The FGR ratio was determined by the number of FGR pups in relation to the total number of pups. Data are presented as mean ± SD.

# *P*<0.05,

* *P*<0.01 *vs* PBS-treated FGR group (*n* = 10).

**Table 3:** Effect of leptin on lung maturity and respiratory function of neonatal rats.

Group	Lung WetWeight (g)	RespiratoryScore	HistologicalScore
Sham control	0.16±0.02#	4.91±0.22#	4.85±0.36#
FGR+PBS	0.14±0.02	4.30±0.75	4.28±0.37
FGR+leptin	0.15±0.02#	4.65±0.47#	4.52±0.39#

After respiratory pattern observation as described in Methods section, an immediate removal of the lungs was performed. The lungs were weighed and then placed in vials containing 10% buffered formalin for histological analysis. Data are presented as mean ± SD,

# *P*<0.05, *vs* PBS-treated FGR group (*n* = 10).

**Figure 3:**
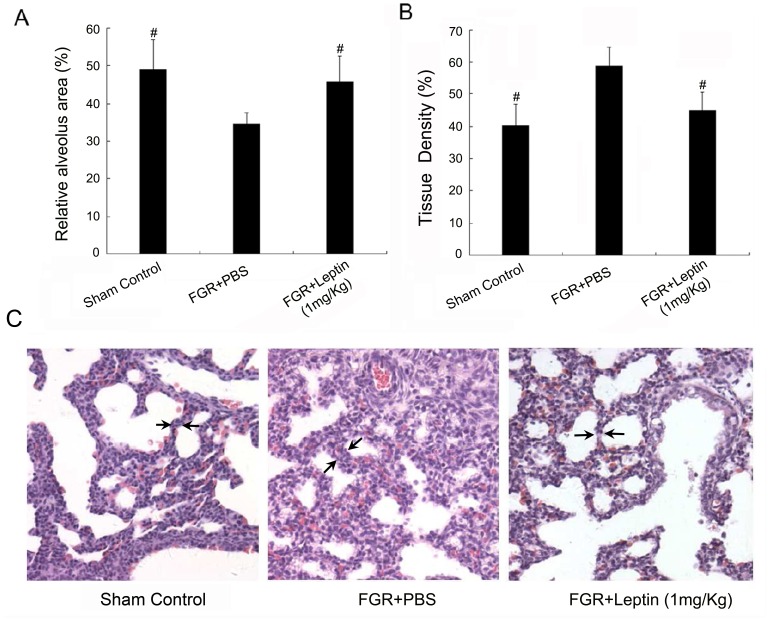
Leptin enhances the maturity of fetal lungs in the FGR rat model. A: The relative alveolus area is presented by the ratio of the alveolar area to the lung area, which was calculated by an image analysis program. *^#^P*<0.05 *vs* PBS-treated FGR group (*n* = 3). B: The field occupied by alveoli and lung tissue is presented as the tissue density, which was analyzed and calculated by ImagePro Plus image analysis software. *^#^P*<0.05 *vs* PBS-treated FGR group (*n* = 3). C: Histological examination of HE-stained lung tissue sections of fetal rats on day 21 of gestation showed a difference in pulmonary alveoli and interval (arrow noted) between the FGR group, leptin-treated group and control (×200).

We also investigated respiratory function and evaluated fetal lung development of pups in the FGR model. As shown in Table 3, enhanced respiration of FGR neonates with leptin treatment was confirmed with respiratory scores and histological scores. Moreover, leptin treatment significantly increased the relative alveolar area and reduced the tissue density in FGR pups (Figure 3A & 3B, *P*<0.05). At high magnification (Figure 3C), we observed that the morphological characteristics of the alveoli and alveolar septal thickness (arrow noted in Figure 3C) in the leptin-treated group were similar to those of the sham control group, suggesting that leptin significantly promoted fetal lung maturity in the FGR rat model.

### Leptin Upregulates the Expression of SP-A in Fetal Lungs and Type-II AECs

**Figure 4:**
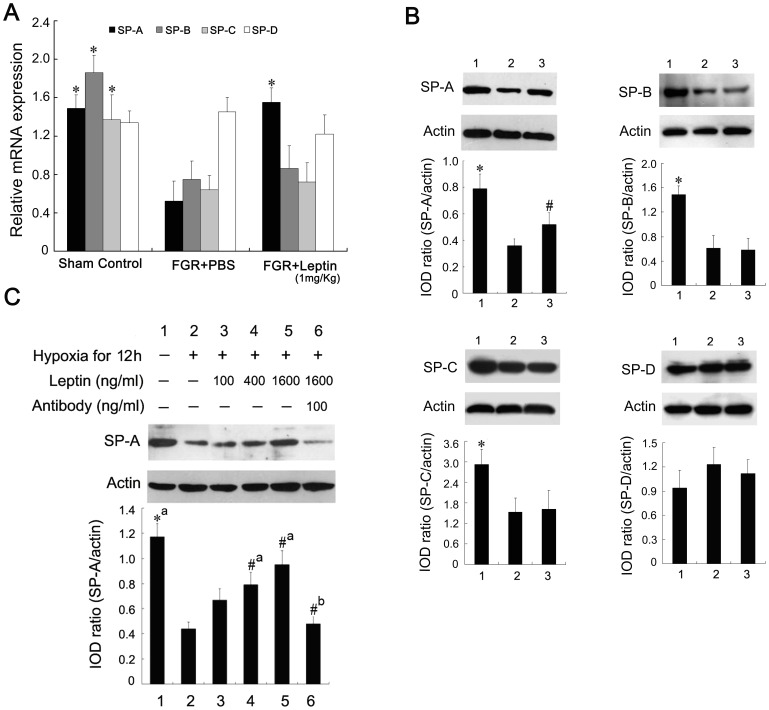
Effect of leptin on the expression of SP-A in fetal lungs and type-II AECs. A: Fetal lung tissue from the sham control group, PBS-treated FGR group, and leptin-treated FGR group were collected and total RNA was extracted for mRNA expression measurement of surfactant protein A (black), B (dark gray), C (light gray) and D (white) by quantitative real-time RT-PCR. ^*^P<0.01 *vs* the PBS-treated FGR group (*n* = 3). B: Fetal lung tissue isolated from the sham control group (lane 1), PBS-treated FGR group (lane 2), and leptin-treated FGR group (lane 3) were subjected to western blot analysis to determine the protein levels of surfactant protein A, B, C and D, which were semi-quantified by densitometry and normalized by β-actin. Data are presented as mean ± SEM. ^*^P<0.01, *^#^P*<0.05 *vs* the PBS-treated FGR group (*n* = 3). C: Cultured type-II AECs were exposed to normal conditions or hypoxia for 12 h with different concentrations of leptin or specific antibody against leptin. Whole lysates were detected by immunoblotting with anti-SP-A antibody. ^*^P<0.01, *^#^P*<0.05; a: *vs* the hypoxia-treated group, b: *vs* the 1600 ng/mL leptin-treated group (*n* = 3).

In this study, we investigated in FGR rats whether leptin modulates the expression of major surfactant proteins, which is the definitive characteristic of type-II AECs. The mRNA level and protein expression of SP-A, SP-B, SP-C and SP-D were determined by quantitative real-time RT-PCR assays and western blotting analysis, respectively. As shown in Figure 4A and 4B, the mRNA and protein of lung SP-A, SP-B, and SP-C, but not SP-D, was lower in FGR than control neonates (*P*<0.01). However, there was a significant increase in mRNA level and protein expression of SP-A, but not SP-B and SP-C, in FGR rats with leptin treatment. This effect of leptin on SP-A expression was also examined in cultured type-II AECs. Western blotting showed that leptin treatment markedly upregulated SP-A expression in type-II AECs cultured under hypoxic conditions. Densitometric measurement of bands showed that SP-A protein levels in cultured type-II AECs treated with leptin were increased in a dose-dependent manner (Figure 4C, *P*<0.05). To further define the positive effect of leptin on SP-A expression, we determined whether an antibody against leptin blocked SP-A expression induced by leptin *in vitro*. Treatment with the antibody at 100 ng/ml downregulated the protein level of SP-A *in vitro*, and protein levels in the antibody-treated group were significantly lower than those in the leptin-induced group (Figure 4C, *P*<0.05). These results suggested that leptin promoted fetal growth and fetal lung maturity in the FGR rat model partially through upregulating the expression of SP-A in type-II AECs.

### Leptin Exerts a Positive Effect on TTF-1 Expression in Type-II AECs

**Figure 5:**
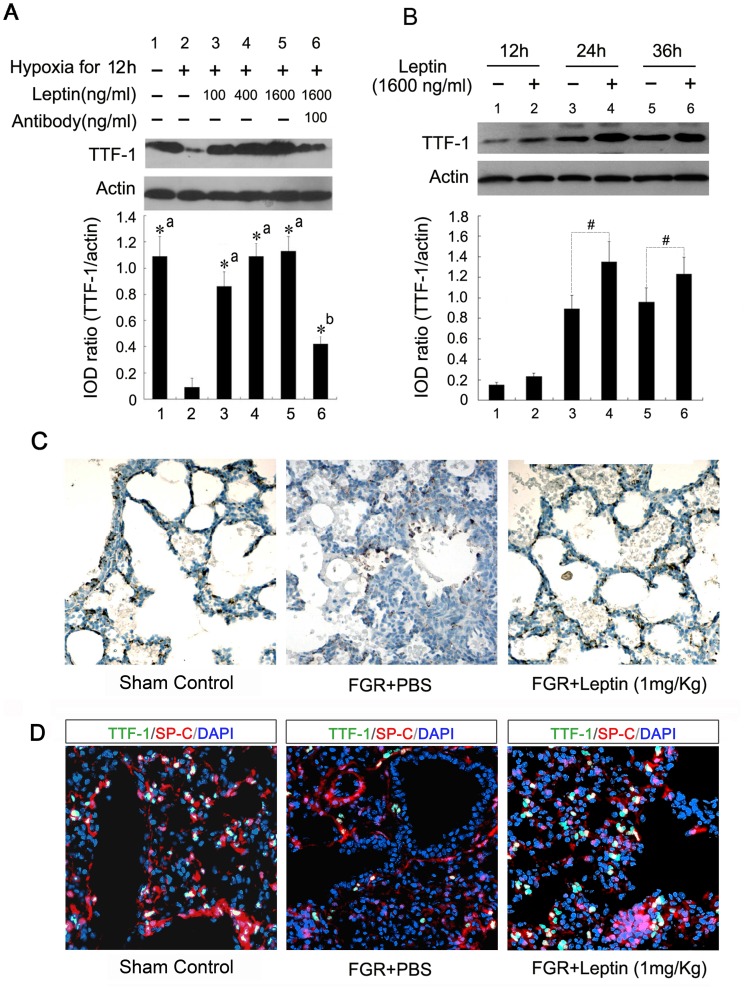
Effect of leptin on the expression of TTF-1 in fetal lungs and type-II AECs. A: Cultured type-II AECs were treated as above, and whole lysates were detected by immunoblotting with TTF-1 antibody. ^*^P<0.01; a: *vs* the hypoxia-treated group, b: *vs* the 1600 ng/mL leptin-treated group (*n* = 3). B: Type-II AECs were exposed to hypoxia for 12 h and treated with 1600 ng/ml leptin for the indicated times. Protein levels were analyzed by immunoblotting. *^#^P*<0.05 *vs* the corresponding untreated cells (*n* = 3). C: Lung tissues from fetal rats at 21 days’ gestation were fixed and incubated with a monoclonal antibody against TTF-1, and visualized by diaminobenzidine color reaction (×200). TTF-1 was mainly expressed in the nuclei of AECs, and leptin markedly upregulated the expression of TTF-1 in the lungs of FGR fetuses. D: Immunofluorescent analysis with antibodies against TTF-1 (green) and SP-C (red) showed that TTF-1 mainly expressed in the type-II AECs and increased in fetal lungs of FGR rats with leptin treatment. Nuclei were stained with DAPI (blue) (×320).

TTF-1 is a key transcriptional factor in lung development and epithelial cell differentiation [27], [28]. Therefore, the effects of leptin on protein levels of TTF-1 in primary cultured type-II AECs were investigated. The protein expression of TTF-1 in type-II AECs was downregulated under hypoxic conditions for 12 h compared with the control group (Figure 5A, *P*<0.01). This change in TTF-1 expression was reversed to a certain extent by leptin in a dose-dependent manner. Moreover, the positive effect of leptin on TTF-1 expression was further defined by the anti-leptin antibody. TTF-1 protein levels were significantly downregulated by the antibody at a concentration of 100 ng/ml compared with the leptin-induced group in an *in vitro* system (Figure 5A, *P*<0.01). The results of western blot assay further confirmed that leptin upregulated TTF-1 expression in a time-dependent manner (Figure 5B). This positive effect was also examined in the FGR rat model. The type-II AECs were identified by staining with anti-SP-C antibody *in vivo*. The TTF-1 protein was mainly expressed in the nuclei of type-II AECs in rat lung tissue as shown in Figure 5C & 5D. Consistent with the *in vitro* results, leptin markedly upregulated the level of TTF-1 in type-II AECs of FGR rats as determined by immunohistochemical analysis (Figure 5C) and indirect immunofluorescence (Figure 5D).These findings suggested that leptin played a positive role in TTF-1 expression in type-II AECs.

### Leptin Enhances the Transcriptional Activity of TTF-1

**Figure 6:**
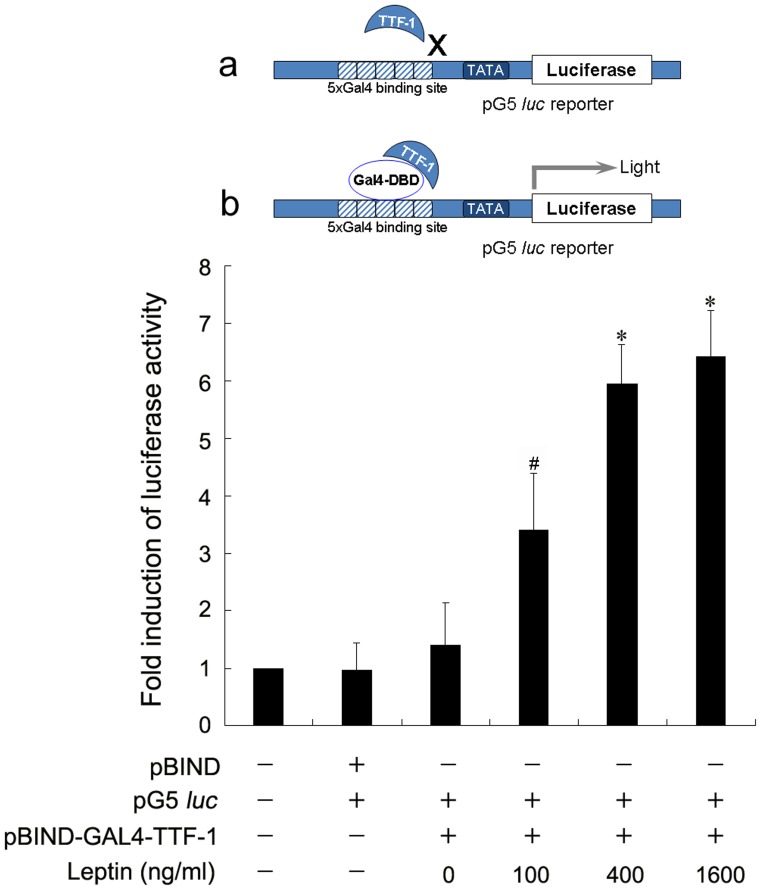
Effect of leptin on the transcriptional activity of TTF-1. A plasmid mixture of pG5, TK-renilla reporter constructs, and pBIND-GAL4-TTF-1 were transfected into A549 cells. After transfection for 6 h, the A549 cells were further cultured with the indicated dosage of leptin for 24 h. The cells were lysed and luciferase activity was measured and normalized by renilla luciferase activity. Results are shown as fold induction in relation to the activity in controls. ^*^P<0.01 *vs* the control cells (*n* = 3).

We next investigated whether the transcriptional activity of TTF-1 protein could also be regulated by leptin. The alveolar epithelial cell line A549 was used in transcriptional activity analysis instead of type-II AECs for higher transfection efficiency. To avoid interference from the endogenous TTF-1 expression induced by leptin treatment, TTF-1 protein was fused in frame with the DNA binding domain of the yeast TF GAL4. The fusion protein pBIND-GAL4-TTF-1 could bind to the luciferase reporter vector pG5 by the five tandem GAL4 binding sites, and the endogenous TTF-1 protein, lacking the DNA binding domain of GAL4, was unable to activate pG5 (Figure 6). Leptin dose-dependently enhanced TTF-1 transcriptional activity (Figure 6). Taken together, these results suggest that leptin is involved in the regulation of TTF-1 expression, as well as transcriptional activity.

### Leptin-induced SP-A Expression is TTF-1 Dependent in Type-II AECs

**Figure 7:**
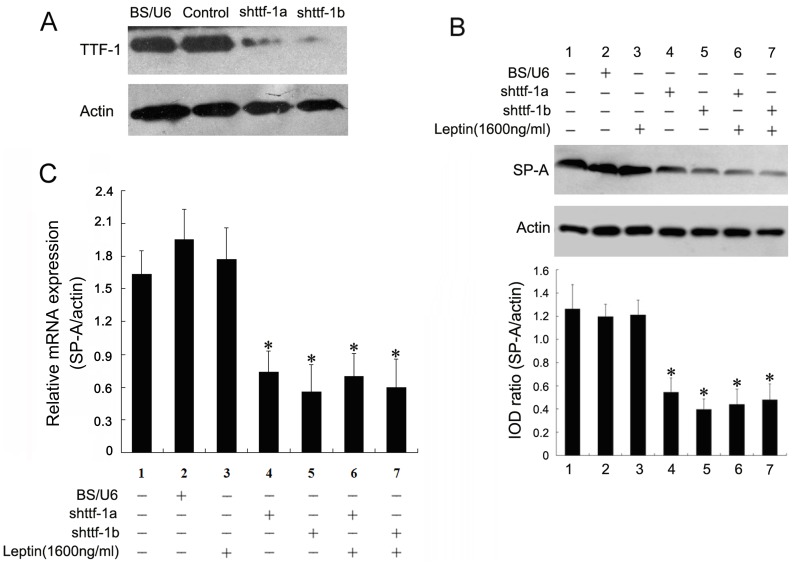
Blocking TTF-1 expression attenuates leptin-mediated SP-A upregulation. A: The gene expression of TTF-1 was knocked down by shRNA, and confirmed by western blotting with TTF-1 antibody. B: Type-II AECs were transfected with the plasmids described above by Lipofectamine 2000, and after 6 h, the transfected cells were exposed to leptin for 24 h. Cell lysates were harvested and the silencing efficiency was verified by western blotting. ^*^P<0.01 *vs* the control group (*n* = 3). C: Type-II AECs were treated as in (B) and total RNA was extracted for mRNA expression measurement of SP-A by quantitative real-time RT-PCR. ^*^P<0.01 *vs* the control group (*n* = 3).

As described above, the gene expression of SP-A was regulated by TTF-1 and leptin in pulmonary epithelial cells. Therefore, with regard to the positive effect of leptin on TTF-1, we hypothesized that upregulation of SP-A expression induced by leptin is associated with TTF-1. To test this hypothesis, two shRNAs targeting TTF-1 were constructed for assessing the effects of TTF-1 on the leptin-induced gene expression of SP-A. We observed that both shRNAs inhibited >80% of the gene expression of TTF-1 in type-II AECs (Figure 7A). We further evaluated the effect of shRNA targeting TTF-1 on the expression of SP-A in type-II AECs. After transfection of the interference plasmids for 24 h, we found that SP-A expression in type-II AECs was inhibited compared with that in the sham control groups (Figure 7B, *P*<0.01). Additionally, co-treatment of leptin and shRNAs targeting TTF-1 was investigated. The upregulation of SP-A induced by leptin was not present with TTF-1 shRNA administration (Figure 7B). This effect was also similar for mRNA expression of SP-A in type-II AECs (Figure 7C). These data suggest that leptin-induced SP-A expression may be TTF-1 dependent in type-II AECs.

## Discussion

The principal findings of this study relate to the molecular mechanisms by which leptin promotes lung maturity in the fetus. We observed that leptin was a potent stimulator of prenatal lung development, and its application in late pregnancy could effectively promote fetal growth. Leptin improved the maturity of fetal lungs and upregulated SP-A expression by a TTF-1-dependent mechanism in type-II AECs.

A strong statistical link between poor fetal growth and pulmonary morbidity has been shown in epidemiological studies [36]. Some studies have suggested an increased risk of acute or chronic pulmonary conditions in FGR infants, such as respiratory distress syndrome, neonatal asphyxia, and bronchopulmonary dysplasia, which are mostly related to fetal pulmonary immaturity and a lack of surfactant [2], [3], [4]. Clinical application of glucocorticoids has been used as a prenatal treatment to induce fetal maturation with specific effects on the fetal lungs since 1972 [5], [6], [37]. However, fetal exposure to excess glucocorticoids has been proposed as a core mechanism underlying the associations between birth size and later disease risk [38], [39]. Studying potentially new growth factors in intrauterine and neonatal development is increasingly being recognized as important and necessary.

Several studies have demonstrated that the cytokine leptin sharply increases in human fetal lungs from approximately 25 wk of pregnancy and reaches its highest levels when the rate of surfactant accumulation is maximal. Leptin receptors are expressed in placental and fetal tissues, suggesting a role for leptin in placental and/or fetal growth [40], [41]. Additionally, blood concentrations of leptin in adulthood may be related to body weight at birth, independent of adult adiposity [42]. In the current study, we showed that protein expression of leptin and Lep-R gradually increased in fetal lungs of the late gestation rat, and it was significantly downregulated in fetal lungs of the FGR rat. These results are consistent with previous studies, which demonstrated that leptin plays a major role in pulmonary epithelial cell differentiation and lung development [8], [10]. In addition, the present study showed that leptin treatment positively affected the body weight of fetal rats and promoted fetal growth in the FGR rat model. Microscopically, we showed that leptin treatment improved lung structure in FGR rats, with a more consummate alveolar structure, larger alveolar cavities, and thinner alveolar walls compared with the sham control group. Previous studies have shown that leptin affects the adult respiratory system by regulating lipid metabolism [43], [44]. The current study demonstrates that leptin may be a multifunctional growth factor for the lung, not only by playing a part in adult lungs, but also by regulating fetal lung development and improving lung structure. This suggests that leptin has great therapeutic potential in the treatment of FGR.

Leptin may act on the epithelium at the Lep-R in alveolar type-II pneumocytes and induce the expression of pulmonary surfactant proteins such as SP-A, SP-B and SP-C. As shown in Figure 4, the protein expression of SP-A not SP-B or SP-C was significantly upregulated in FGR rats with leptin treatment. SP-A is required for tubular myelin formation and plays a regulatory role in maintaining the structure and function of pulmonary surfactant complex [25], [26]. SP-A is primarily synthesized by type-II AECs, and is developmentally regulated in the fetal lungs in concert with surfactant glycerophospholipid synthesis [23]. The actions of leptin to promote fetal lung maturation and surfactant synthesis appear to be species-specific. A recent study concluded that leptin does not affect surfactant levels in fetal sheep and mice [45]. It seemed to bring up a contradiction with the results from previous studies, which demonstrated that leptin enhanced lung maturity and upregulated surfactant components in fetal rats [8]. The current study examined the mechanism of how leptin regulates SP-A expression in respiratory epithelial cells. Using a model of type-II AECs induced by hypoxia, we demonstrated that leptin upregulated SP-A expression and this was released in a dose-dependent manner. A special antibody against leptin blocked the expression and release of SP-A *in vitro*. *In vivo*, leptin increased the production of SP-A in FGR fetal lungs. These results indicated that leptin improved fetal lung maturity and promoted fetal growth in the FGR rat model, partially by upregulating the expression and secretion of SP-A in type-II AECs. We also found that TTF-1, a transcriptional factor, was involved in leptin-induced SP-A expression, which may be partially responsible for the promotion of fetal lung maturity and fetal growth in the FGR rat model with leptin treatment.

Pulmonary TTF-1 plays a critical role in branching morphogenesis of the peripheral lung and the expression of surfactant proteins such as SP-A, SP-B and SP-C [28], [46], [47]. Interestingly, in the present study, we showed that leptin antagonized the decrease in TTF-1 protein induced by hypoxia. This effect was dose-dependent, but was reduced by adding leptin antibodies *in vitro*. Leptin markedly upregulated the level of TTF-1, which was mainly expressed in the nuclei of alveolar epithelial cells. Moreover, data of the luciferase reporter assay showed that leptin remarkably enhanced TTF-1 transcriptional activity. The upregulation of SP-A induced by leptin was not present after TTF-1 shRNA administration, suggesting that leptin-induced SP-A expression may be TTF-1-dependent in type-II AECs. Surprisingly, no significant effect of leptin on SP-B or SP-C expression was observed in the in vivo experiments and more research will be required to illustrate the comprehensive effects of leptin on the expression of SP-B and SP-C by the network of transcription factors including TTF-1.

The results of this study clearly showed positive effects of leptin on lung development in the fetus by increased expression of SP-A in type-II AECs. The transcriptional factor TTF-1 is involved in leptin-induced lung maturity and SP-A expression, which may represent a novel biological activity of leptin. Further investigation into the molecular mechanism of leptin in the regulation of TTF-1 expression and transcriptional activity will allow for the development of more potential candidates for the treatment of FGR.
